# The Impact of Surgical Continuity of Care on Postoperative Outcomes and Hospital Readmissions: A Review

**DOI:** 10.7759/cureus.90207

**Published:** 2025-08-16

**Authors:** Mario Antonio Villatoro Bonilla, Cristian Israel Sarmiento Bonilla, Maria Fernanda Desirée Rojas Herrera, Felgir Josué Noé Herrera Palacios, Mauricio David Ramírez Pineda, Manrique Vega Solano

**Affiliations:** 1 Surgical Oncology, Instituto Salvadoreño del Seguro Social, San Salvador, SLV; 2 Surgery, Hospital de Especialidades UMAE (Unidad Médica de Alta Especialidad) No. 71, Torreón, MEX; 3 Faculty of Medical Sciences, Universidad de San Carlos de Guatemala, Guatemala City, GTM; 4 Health Policy, Universidad de las Américas, Quito, ECU; 5 Emergency Medicine, Vegas Medical Group, San José, CRI

**Keywords:** impact, literature review, postoperative outcomes, s: hospital readmissions, surgical continuity of care

## Abstract

This review stems from the rising fragmentation of surgical care as care tends to get transferred among several teams and between several providers, leaving patients confused and unsatisfied. This fragmentation can lead to worse clinical outcomes, especially among high risks surgery patients. The review aims to examine how continuity of surgical care affects recovery, complication management, and patient satisfaction. Comprehensive discharge planning, individualized patient education, timely follow-up (especially with the operating surgeon or through home visits by familiar providers), and the integration of Advanced Practice Providers can substantially reduce readmissions. While common, follow-up phone calls alone were not consistently sufficient to reduce readmissions. Care fragmentation, particularly the absence of surgeon care continuity during readmission, has been independently associated with increased 30-day mortality and a longer time to receive necessary interventions. Technological interventions like telemedicine offer promising avenues for remote monitoring, education, and consultation, leading to improved patient satisfaction, reduced costs, and lower readmission rates across various surgical specialties. Additionally, peer mentoring programs can effectively reduce patient anxiety and facilitate earlier communication of complications, even if this results in an increase in beneficial early readmissions. Enhanced Recovery After Surgery (ERAS) protocols have also demonstrated effectiveness in shortening length of stay and improving recovery. This review reinforces the critical importance of understanding and improving SCOC as a cornerstone of patient-centered care. By bridging the gap between hospital discharge and home recovery, effective SCOC interventions can demonstrably improve patient outcomes, reduce burdensome readmissions, enhance patient satisfaction, and elevate overall quality of life. The complexity of the concept of continuity of care, affecting clinical, informational, and relational dimensions, pinpoints the necessity of the join and concerted efforts at the healthcare continuum.

## Introduction and background

The new model of care to enhance outcomes of patients, especially those who have undergone surgical intervention, is continuity of care. The purpose of the model is to offer continuous and coordinated healthcare advice to the patients once they leave the hospital, enhance and encourage physical rehabilitation, and enhance quality of life [1]. Continuity of care may help prevent poor clinical diagnosis and treatment. It also eliminates postoperative complications and improves the clinical outcomes of patients [2]. In addition, developed countries with extensive adoption of continuity of care systems in different surgical specialties, including cervical cancer, colorectal surgery, craniotomy, new ileostomy, coronary artery bypass graft (CABG) valve replacement and valve surgery, pancreatectomy, open ventral hernia repair, general surgeries, and vascular surgeries, exhibited good and encouraging responses to enhance patient outcomes and minimize the readmission rate [3-7]. It was also indicated in the literature findings that well-structured post-discharge follow-up, communication, and coordination amongst the health providers enhance the clinical outcome of the patient, decrease the number of hospital readmissions, and improve the quality of life [8,9].

A significant challenge in the modern healthcare system is the high rate of hospital readmission, specifically in surgical settings. According to data from 2014, over 2,500 hospitals in the United States were penalized for high 30-day readmission rates under the Affordable Care Act, indicating poor continuity of care [10]. A realistic, cost-effective readmission reduction approach is needed to reduce financial penalties and improve treatment quality [10]. Center for Medicare and Medicaid Services (CMS) data indicate that 20% of Medicare patients are readmitted within 30 days of release, costing over $17 billion annually [11]. Up to 30% of patients may be readmitted following complex abdominal surgical procedures. Hospital readmission rates are under more critical scrutiny [12,13]. For instance, hospitals must disclose readmission statistics, and those with high readmissions earn lower CMS reimbursement for certain surgical procedures [14]. Therefore, improved transitional or continuity of care and self-monitoring may reduce preventable readmissions for such high-risk populations.

Recently, the literature has been growing with a focus on readmission risk factors. Literature proposed patient-centered care transitions to effectively reduce hospital readmissions [15]. But few studies in the current literature have applied them in the context of surgical settings. For example, a mixed-method, multi-step observational study involving inpatient medicine, geriatrics, and primary care patients found that transitional care processes used and the risk-standardized readmission rate are significantly reduced through identified continuity of care evidence-based processes [16]. Another systematic review and meta-analysis, including 15 primary studies, found that outpatient follow-up visits may minimize 30-day all-cause readmissions for heart failure and stroke patients [17], but the applicability of findings in the surgical care model is limited and underexplored.

The rationale of this review stems from the rising fragmentation of surgical care, as care tends to get transferred among several teams and between several providers, leaving patients confused and unsatisfied. This fragmentation can lead to worse clinical outcomes, especially among high-risk surgery patients [18]. This review will give a reflection on how continuity of care may improve surgical outcomes by reflecting on how structured follow-up will contribute to better surgical care by informing policy regarding value-based care and voice bundled payments. This review would support patient-centered care principles and optimize surgical team structures. This review is conducted to determine if continuity of care after surgery may reduce readmissions across surgical specialties. The authors of this review hypothesized that a coordinated discharge procedure, involving a dedicated healthcare provider such as an Advanced Practice Provider (APP), can prevent readmissions and improve patient-related outcomes such as recovery, complications, and mortality in high-risk patients.

This literature review will explore the significance of readmissions as a critical factor, where postoperative complications are managed by surgical continuity of care, and its influence on patient outcomes. It will examine how continuity of surgical care affects recovery, complication management, and patient satisfaction compared to fragmented care handed over to different surgeons or teams. The topic is timely, given the increasing pressure to measure surgical outcomes and optimize continuity under bundled payment and value-based care systems.

## Review

**Figure 1:**
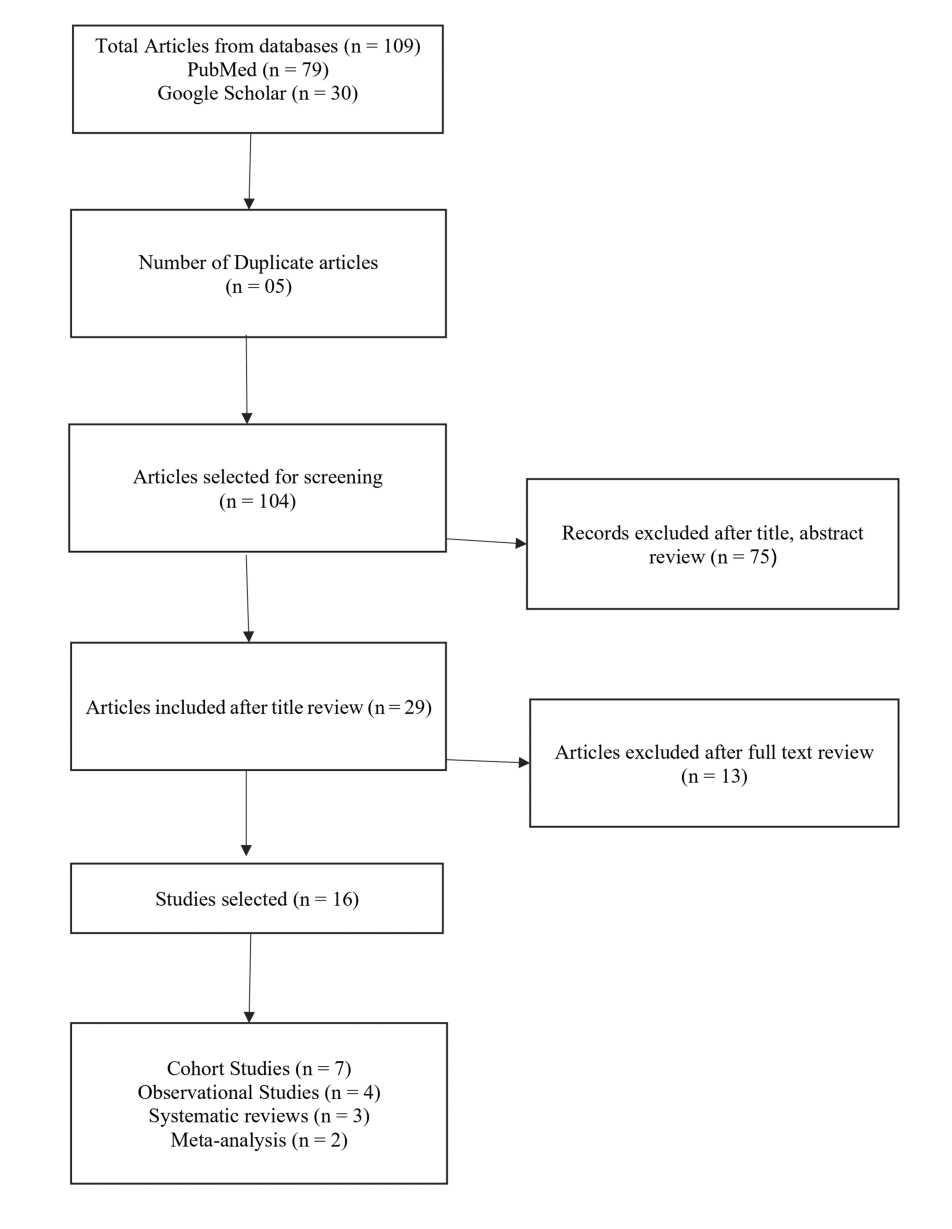
Flow shart of the study selection process

This literature (narrative) review was conducted using relevant topic-specific keywords such as “Surgical Continuity of Care”, “Continuity of Care”, “Continuity of Service”, “Continuum of Care”, “Continuity of Surgical Care and Patient Outcomes", “Transitional Care”, “Surgical Outcomes”, “Surgeon-Patient Relationship”, “Same-Surgeon Care”, “Complication”, Reoperation Rates”, “Patient Satisfaction”, “Hospital Readmission Rates”, “Postoperative Management by Operating Surgeon”, “Surgical Continuity and Hospital Readmission”, and “Impact of Surgeon on Continuity of Surgical Care”. Boolean operators were used to incorporate the keywords and search on PubMed and Google Scholar, focusing on articles published between January 2015 and June 2025. The search was limited to peer-reviewed literature in the English language. The effectiveness of surgical continuity of care was determined by extracting data from included studies, including author, year, study design, surgical procedure, surgical continuity of care, clinical outcomes, hospital readmission rates, and significant outcomes (p < 0.05). The study selection process is illustrated in Figure 1. 

The narrative review included 16 studies (seven cohort studies, four observational studies, three systematic reviews, and two systematic reviews and meta-analyses) between January 2015 and June 2025. The characteristics of the included studies are presented in Table 1.

**Table 1:** Characteristics of reviewed studies RCTs: Randomized Controlled Trials, CABG: Coronary Artery Bypass Grafting, TAAA: Thoracoabdominal Aortic Aneurysm, NPs: Nurse Practitioners, PCP: Primary Care Physician, SPARCS: New York Statewide Planning and Research Cooperative System, VOPC: Virtual Outpatient Clinics, OPC: Outpatient Clinics, LT: Liver Transplantation, THMP: Telemedicine-based Home Management Program, SOC: Standard of Care, QOL: Quality of Life, VIPAR: Virtual Interactive Presence and Augmented Reality, TME: Telemedicine Encounter, KT: Kidney Transplant, ERAS: Enhanced Recovery After Surgery, LOS: Length of Stay, PPR: Patient-Perceived Readiness, OG: Observation Group, CG: Control Group, THA: Total Hip Arthroplasty, TKA: Total Knee Arthroplasty, WMD: Weighted Mean Difference, OR: Odds Ratio, SMD: Standardized Mean Difference, CI: Confidence Interval, VHA: Veterans Health Administration, RSRRs: Risk-Standardized 30-Day Readmission Rates, APP: Advanced Practice Provider, FTR: Failure to Rescue

Author, Year	Study Design	Population Characteristics	Intervention Program	Surgical Procedure	Outcomes	Findings (p < 0.05)
Jones et al., 2016 [19]	Systematic Review (3 RCTs, 2 Prospective Cohorts, 5 Retrospective)	15072 patients, 7536 in the intervention group, and 7536 in the control group.	Discharge planning program, Follow-up phone calls, Patient education, Home visits by specialized nurse practitioners (NPs) and physician assistants, Surgeon postoperative follow-up visit, and PCP follow-up.	CABG (valve replacement), colectomy, craniotomy, new ileostomy, pancreatectomy, open TAAA, open ventral hernia repair, general surgeries, and vascular surgeries.	Transitional care interventions and their effect on hospital readmissions after surgery.	Discharge planning programs, patient education interventions, primary care follow-up, and home visits reduced readmissions by 11.5% (P = .001), 23.5% (P < .05), 8.3% (p < 0.001), and 7.69% (P = 0.023), respectively.
Missios and Bekelis, 2016 [20]	Cohort Study	16,483 spine surgery patients	Outpatient Continuity of care, such as New York Statewide Planning and Research Cooperative System (SPARCS)	Spine surgery	30-day post-discharge readmission	10.9% readmitted rate among patients who were evaluated, where the original surgery was performed as compared to 12.0% readmitted rate (different hospital evaluation).
Bekelis et al., 2016 [21]	Cohort Study	452 cerebral aneurysm patients	Surgical Continuity of Care, such as New York Statewide Planning and Research Cooperative System (SPARCS)	Cerebral aneurysm surgery, such as surgical clipping or endovascular coiling	To investigate association of 30-day readmissions with evaluation in the hospital where the original procedure was performed	The original hospital was associated with a decreased rate of 30-day readmission (OR=0.41; 95% CI 0.22 to 0.78), indicating that assessment at the original hospital had a lower readmission rate.
Justiniano, et al. 2019 [7]	Cohort Study	20,016 colorectal surgery patients (13,344 intervention, 6,672 control group)	Telemedicine-based continuity of care, such as the Statewide Planning and Research Cooperative System	Colorectal surgery	Post-discharge continuity of care, defined at the hospital and surgeon level, was associated with a decreased mortality rate after colorectal surgery.	12.4% had neither hospital nor surgeon care continuity. Absence of care continuity (surgeon & hospital) increased the risk of 30-day mortality by 2.04 times (95% CI: 1.72 to 2.42) and by 2.65 times (95% CI: 2.18 to 3.30).
Healy et al., 2019 [22]	Randomised controlled trial	209 patients, 107 virtual outpatient clinics (VOPC), 102 actual outpatient clinics (OPC)	Telemedicine-based monitoring at home via VOPC	General surgical services such as minor surgery or elective or emergency surgery such as appendectomy, hernia repair, thyroidectomy, head injury, cellulitis, colonic polyps, or Barrett’s esophagus.	to establish whether VOPC was an acceptable alternative to OPC attendance for a broad range of general surgical patients following a hospital admission.	83% in the VOPC group and 67% in the OPC group preferred and were satisfied with a VOPC appointment as their future follow-up of choice (p = 0.029).
Lee et al., 2019 [23]	Randomized controlled trial	101 LT patients, 50 in the TH, 51 in the group, and 51 in the Standard of Care (SOC) group	The THMP included an electronic tablet and Bluetooth devices to support daily text messages, education videos, and video FaceTime capability; data was cyber-delivered into our electronic medical record daily.	Liver Transplantation (LT)	To assess the impact of THMP on patient adherence, hospital readmissions, and quality of life (QOL) after LT.	The THMP group had a lower 90-day readmission rate compared to SOC (28% vs 58%; P = 0.004) and improved QOL in regard to physical function (P = 0.02) and general health (P = 0.05) at 90 days.
Huang et al., 2019 [24]	Narrative Literature Review	54 primary studies	VIPAR, Telerobotics, or direct surgical intervention, and telementoring, surgeon to surgeon, teleconsulting, surgeon to other specialists or primary care physicians, Telemedicine for postoperative follow-up and tele-educating, especially in resource-limited regions.	Neurosurgery (41), Orthopedic Surgery (79), General Surgery (180), Plastic Surgery (126), Cardiac Surgery (36), Ophthalmology (121), Urology (44).	Telemedicine-based role in post-op follow-up & communication across surgical teams.	Telemedicine is used successfully to care for patients in remote areas.
Hogikyan, et al. 2021 [6]	Cohort Study	17 patients	Significance of the surgeon-patient relationship on surgery care	Otolaryngology head and neck surgery	To qualitatively elucidate the mechanisms of how trust develops between otolaryngologists and their patients.	Surgeon trust is valued by patients. Optimal surgical care paradigms should foster meaningful preoperative surgeon-patient connections and favorable surgeon and institutional reputations to build confidence.
Chun et al., 2022 [25]	Randomized controlled trial	80 patients Peer Intervention group = 40, control group = 40	Peer mentoring program	Kidney transplant (KT)	Investigate the impact of the program on patient anxiety and 30-day readmissions.	The treatment arm experienced a 3.42-point greater decrease in anxiety score over 30 days, suggesting that care transitions using mentors decrease anxiety levels but increase readmission due to early identification of complications leading to timely treatment.
Diaz-Miron et al., 2022 [26]	Prospective, observational survey study	73 surgeons; Early pandemic perception survey, Perception after two months of pandemic	Adoption of TME as care of patients	Pediatric general, neurosurgery, orthopedic, otolaryngology, plastic surgery, and urology.	To evaluate surgeon and caregiver perspectives of TMEs during the pandemic.	Both groups had little pre-pandemic TME experience, although it was positive. Positive TME experiences may stimulate continued use for patient care.
Min et al., 2023 [27]	Randomized controlled trial	52 patients, Exercise group (EG) = 26, Usual care group (UC) = 26	Exercise as ERAS program	Colorectal surgery	Length of stay (LOS), patient-perceived readiness (PPR) for hospital discharge, and physical function.	Median LOS of EG = 6 days, UC = 6.5 days (P = 0.021). Increase of 0.63 kg muscle mass in EG than UC, p = 0.03, and a mean difference of 14.4 for PPR, p < 0.001 for EG.
Zhang et al., 2024 [3]	Retrospective observational study	257 children, Observation group (OG) = 108, control group (CG) = 149	Internet Family Engagement Continuum of Care via mobile medical platform.	Strabismus ambulatory surgery	Impact of the program on postoperative complications, readmission rate, children’s quality of life, and parental satisfaction.	In OG, the readmission rate (10.19%) and complication incidence (6.48%) were significantly lower than in CG (22.82%) (P = 0.009) & (18.79%) (P = 0.005), respectively.
Zhang et al., 2024 [4]	Systematic review and meta-analysis	76 971 patients, ERAS group = 29 702, control group = 47 269	ERAS interventions	THA or TKA	To evaluate the significance of ERAS interventions for postoperative outcomes.	ERAS could significantly shorten the LOS (WMD = -2.65, P < .001), reduce transfusion rate (OR = 0.40, P < .001), and lower 30-day postoperative mortality (OR = 0.46, P = .01) without increasing postoperative complications or readmission rate.
Fu et al., 2024 [5]	Systematic review and meta-analysis	1687 patients, Intervention group = 845, Control group = 842	Continuity of care model (post-discharge follow-up and enhancing communication and collaboration)	Cervical cancer surgery	To analyze the effect of implementing continuity of care for postoperative patients.	Extended care improved patients' QOL (SMD=1.35, 95% CI (1.05, 1.64), P<0.05), alleviated patients' postoperative anxiety (SMD=-0.92, 95% CI (-1.85, 0.00), P<0.05), and postoperative depression (SMD=-1.15, 95% CI (-1.35, -0.95), P<0.05).
Sarrazin et al., 2024 [28]	Retrospective cohort study	108 265 patients in VHA and Non-VHA	Surgical continuity of care (Surgical Quality Improvement Program)	Cardiac surgery such as CABG.	Readmissions to acute care VHA or non-VHA hospitals are risk-standardized 30-day readmission rates (RSRRs).	VHA hospitals whose readmission performance metric improved by including non-VHA readmissions had higher patient volume, higher complexity, and a lower proportion of care outside the VHA. Thus, improving continuity of care may have a paradoxical effect of worsening VHA performance metrics.
Alexander et al., 2025 [29]	Retrospective Cohort study	290 patients, pre-APP group = 191, post-APP group = 99	APP-led structured follow-up protocol	Pancreatic resection	To explore if APP was associated with readmission rates, length of stay (LOS), mortality, and failure-to-rescue (FTR) rates.	The post-APP group had lower Charlson-Comorbidity Index scores (3.8 ± 2.2 vs. 4.6 ± 2.0, P < 0.01), more minimally invasive surgery (11.1% vs. 4.7%, P = 0.04), and more benign pathology indications (40.4% vs. 19.9%, In the post-APP group, 48.5% of patients were seen within 72 hours, with shorter index hospitalization LOS (6.9 ds vs 8.6 ds, P = 0.01), earlier first postoperative visits (4.9 ds vs 10.1 ds, P < 0.01), and shorter readmission time (8.1 ± 5.8 vs 11.3 ± 5.7, P = 0.05) compared to the post-APP group; 30-d mortality (1.0% compared to 4.2%, P = 0.17) and FTR rates (1.6% versus 5.8%, P = 0.28) were reduced but not substantially different.

Surgical continuity of care

Continuity of care in surgical populations encompasses various interventions and approaches aimed at ensuring a smooth transition for patients from hospital discharge to home or other care settings, with the goal of improving patient outcomes and reducing readmissions [12,19]. An overview of continuity of care in surgical populations' outcomes is discussed below.

Impact On Patient Outcomes

Hospital readmissions: Studies demonstrate that effective continuity of care interventions can significantly reduce hospital readmissions. For surgical patients, including CABG-valve replacement, colectomy, craniotomy, new ileostomy, pancreatectomy, open hernia repair, and all general surgeries, Jones et al. concluded that discharge planning programs, patient education, primary care follow-up, and home visits have shown reductions in hospital readmission rates [19]. Specifically, discharge planning programs have reduced readmissions by 11.5-23%, patient education by 14-23.5%, and home visits by 4-7.69% [19].

Bekelis et al. carried out a cohort study for patients undergoing cerebral aneurysm surgery. While evaluation in the emergency room (ER) of the hospital where the original procedure was performed was associated with a decreased rate of 30-day readmission (OR=0.41; 95%CI 0.22 to 0.78), suggesting the importance of continuity of care [21]. Similarly, Missios and Bekelis carried out a study to examine spine surgery-admitted patients and, when evaluated in the ER of the hospital, the authors found that it was linked to a lower 30-day readmission rate [20]. Lee et al. reported that in liver transplant patients, a telemedicine-based home management program was associated with significantly fewer readmissions between 31 and 90 days post transplant, particularly for issues trackable by the monitoring device, such as abdominal pain, fever/sepsis, and blood sugar problems [23].

Reduced mortality: For colorectal surgery patients, Justiniano et al. reported that the absence of surgeon care continuity was independently associated with increased 30-day mortality. This fragmentation was also linked to a longer time to receive necessary percutaneous drainage, which in turn was associated with increased mortality [7]. In pancreatic surgery, integrating APPs with a structured follow-up protocol led to earlier identification of complications and lower mortality to 1.0% in the post-APP group as compared to 4.2% in the pre-APP group [29]. Therefore, it is indicated that continuum of care reduces mortality.

Shorter length of stay (LOS): Min et al. conducted a randomized controlled trial to introduce postoperative exercise programs for colorectal cancer patients that resulted in a shorter median LOS [27]. Similarly, Qingqing et al. conducted a systematic review and meta-analysis on the implementation of Enhanced Recovery After Surgery (ERAS) protocols for osteo-arthroplasty and found that ERAS was associated with a significantly shorter hospital LOS [4].

Improved patient readiness and satisfaction: Min et al. observed that a postoperative exercise program enhanced patients' perceived readiness for hospital discharge among colorectal cancer patients (Mean difference, MD = 14.4, p < 0.001) [27]. Healy et al., in their RCT, observed that virtual outpatient clinics (VOPC) were a satisfactory alternative to in-person follow-up after general surgical discharge, with a majority of 83% of patients expressing preference and greater satisfaction for VOPC as their future follow-up of choice (p = 0.029) [22].

Intervention programs of SCOC and key components

Comprehensive Discharge Planning

Programs with comprehensive checklists or designated medical professionals can reduce readmissions. In their systematic review, Jones et al. found that implementation of a comprehensive discharge plan for elderly cardiac surgery patients, managed by a nurse, demonstrated a reduction in readmission rates at two weeks from 36% to 18% (P = 0.05) [19]. A similar discharge planning project for craniotomy patients, which involved daily visits from a discharge planner, led to a statistically significant decrease in 30-day readmissions from 17.9% to 5.4% (P = 0.04). The Project RED (Re-engineered Discharge) intervention, which implemented a checklist for discharge planning and included reinforcing follow-up calls, resulted in reduced 30-day readmission rates from 23% to 11.5% (P = .001) following pancreatectomy. These findings highlight how coordinated discharge processes, often driven by a dedicated individual or a structured checklist, can lead to significant reductions in hospital readmissions.

Patient Education

Jones et al., in their systematic review, found that patient-focused, procedure-specific education, initiated early and continued post-discharge, can significantly reduce readmissions, especially for conditions like new ileostomies [19]. Patient education interventions were observed to reduce readmissions by 23.5% (P < 0.05). For instance, an individualized education for cardiac surgery demonstrated a significant reduction in readmission to 1% in contrast to 23.5% of controls who received only a standard education booklet (P < 0.05) at three months post discharge. Furthermore, for patients with new ileostomies, an education program resulted in a reduction of total readmissions from 35.4% to 21.4% (P = 0.28), and notably, readmissions specifically due to dehydration decreased from 15.5% to 0% (P = 0.02). These findings highlight how early and sustained patient-focused education can lead to significant reductions in readmission rates.

Follow-up Communication

Home visits: In their systematic review of transitional care interventions and their effect on hospital readmissions after surgery, jones et al. found that specialized nurse practitioners (NPs) or physician assistants (PAs) conducting home visits were effective in reducing hospital readmissions by 7.69% (P = 0.023), particularly by healthcare providers familiar with the patient's postoperative care [19]. At the same time, Alexander et al. reported that integration of APP-led structured follow-up also led to earlier first postoperative visits (4.9 days vs. 10.1 days, P < 0.01) as compared to the pre-APP group, and these earlier post-op visits by the post-APP group helped to identify patients’ complications early and treat them in a timely manner [29]. Therefore, home visits play a significant role in early identification of complications, which eventually increases surgical patients’ health-related outcomes.

Phone calls: While common, phone calls alone were not consistently independently responsible for reducing readmissions, though they may reduce later readmissions related to comorbidities [19]. Nurse-led telephone follow-up has been found effective in other settings like oncology [22].

Surgeon follow-up: Jones et al. reported that timely follow-up with the surgeon is crucial, especially for patients prone to complications [19]. Similarly, Hogikyan et al. observed that direct surgeon participation in preoperative evaluation and counseling is central to building patient-surgeon trust and relationships [6]. While doing thematic analysis involving 17 surgeons of faculty members, they concluded that surgeon trust is valued by patients. Optimal surgical care paradigms should foster meaningful preoperative surgeon-patient connections and favorable surgeon and institutional reputations to build confidence.

Telemedicine

Telemedicine offers a means for postoperative follow-up and can be a safe and acceptable alternative to traditional clinic visits, with high patient and surgeon satisfaction for simple consultations and straightforward follow-ups [22,24,26]. Telemedicine programs such as the telemedicine-based home management program (THMP) group experienced a significantly lower 90-day hospital readmission rate of 28%, compared to 58% in the standard of care (SOC) group (P = 0.004). The largest difference in readmission rates occurred between days 31 and 90 post-transplant, with the THMP group showing 4% readmission compared to 22% for SOC (P = 0.01). The THMP group also had fewer readmissions for issues trackable by the tablet, such as abdominal pain, fever/sepsis, and blood sugar problems, compared to the SOC group [23]. These findings suggest that telemedicine interventions, by offering closer monitoring and convenient access to the care team, can effectively reduce readmissions and enhance patient quality of life after major surgical procedures such as liver transplantation.

Advanced Practice Providers

Integration of APP with structured follow-up protocols can lead to earlier complication identification, shorter LOS, and lower mortality. Alexander et al. carried out a retrospective cohort study in which 290 patients were enrolled, 191 pre-APP and 99 post-APP. The cohort averaged 65.5 ± 13.4 years old, with 57.2% receiving pancreaticoduodenectomy. Compared to the pre-APP group, the post-APP group had lower Charlson-Comorbidity Index scores (3.8 ± 2.2 vs. 4.6 ± 2.0, P < 0.01), more minimally invasive surgery (11.1% vs. 4.7%, P = 0.04), and more benign pathology indications (40.4% vs. 19.9%, P < 0.05). In the post-APP group, 48.5% of patients were seen within 72 hours, with shorter index hospitalization LOS (6.9 days vs 8.6 days, P = 0.01), earlier first postoperative visits (4.9 days vs 10.1 days, P < 0.01), and shorter readmission time (8.1 ± 5.8 vs 11.3 ± 5.7, P = 0.05) compared to the pre-APP group. In the post-APP group, 30-day mortality (1.0% compared to 4.2%, P = 0.17) and FTR rates (1.6% versus 5.8%, P = 0.28) were reduced but not statistically significant [29].

Peer Mentoring

Chun et al. implemented a standardized peer mentoring program for kidney transplant patients and found it effective in decreasing patient anxiety. Interestingly, it also led to an increase in early 30-day readmissions, but post-hoc analysis revealed these were often beneficial, enabling earlier identification of issues and communication with care coordinators, potentially preventing more severe complications later [25]. This highlights that not all readmissions are negative outcomes and that peer mentors can foster patient communication and self-care.

ERAS Protocol

ERAS is a multidisciplinary protocol that is designed to improve pre-, peri-, and postoperative recovery and can significantly reduce hospital LOS. Zhang et al. reported that ERAS could significantly shorten the LOS (WMD = -2.65, P < 0.001), reduce transfusion rates (OR = 0.40, P < 0.001), and lower 30-day postoperative mortality (OR = 0.46, P = 0.01) without increasing postoperative complications or readmission rate [4].

Challenges and considerations

Studies using administrative data may have miscoding errors or indication bias [7]. Observational studies and cohorts cannot definitively establish causality [3,26]. Relying solely on 30-day readmission rates as a quality metric may not capture the full value of care, as some readmissions can be beneficial for managing long-term care [25]. Factors outside surgical practitioners' control, such as social support or insurance status, can influence readmission likelihood [28]. Not accounting for readmissions occurring in non-Veterans Health Administration (VHA) hospitals can distort hospital performance metrics. For example, in VHA hospitals, one in five readmissions for elderly surgical patients occurred outside VHA facilities, and including these altered nearly half of the hospitals' performance rankings [28]. Despite the benefits of telemedicine, challenges include the need for interpreter services, stable internet connection concerns, user-friendly platforms, and potential “digital divides” where audiovisual satisfaction is higher among higher-income patients [26]. Some patients may also have “app literacy” issues [23]. Long-term reimbursement for telemedicine and multi-state licensing requirements are ongoing concerns for surgeons [26]. Insurance companies may have specific guidelines for how telemedicine visits are logged and billed [24]. 

The lack of a physical examination remains a limitation of phone calls and a barrier to broader telemedicine adoption for some follow-up scenarios [19,28]. The interpersonal connection with a surgeon is paramount to trust development. Prior knowledge of the surgeon or institution and observed team dynamics can influence trust, suggesting that continuity of care in who provides preoperative counseling and who performs the surgery could be important [6].

The issue of continuity of care is multi-dimensional, as it further develops with changes in technological solutions and a better insight into the needs of patients. Innovations and new strategies are instrumental in enhancing patient outcomes and optimizing the postoperative management process. Technology is being seen to play an important role in increasing SCOC. Telemedicine is another major innovative practice that has incorporated telecommunications in the diagnosis and treatment of patients without having to be physically present, irrespective of geographical boundaries [24]. It includes telerobotics and telementoring, which allows experts and surgeons to help them by guiding them through various geographical locations, facilitating knowledge transfer, and addressing disparities in access to surgical care [24]. Teleconsulting, which enables collaboration among specialists such as surgeons and primary care physicians to provide multidisciplinary healthcare, especially for resource-limited areas and rural areas [24]. 

Telemedicine for postoperative follow-up is a key area for SCOC. It allows for continuous monitoring of patients after discharge, addressing the increased risk of complications that are often diagnosed late. Benefits include access to specialty care in rural or medically underserved areas and reduced costs for both patients (e.g., less time off work and reduced travel distance and time) and healthcare systems, such as liberated clinic appointments, decreased unnecessary hospital transfers, and reduced readmissions. Examples include smartphone applications for monitoring surgical wounds and detecting surgical site infections, and remote examination for stoma care [24]. Studies on telemedicine for postoperative care report high levels of satisfaction from both patients and physicians [22,26]. For instance, VOPCs have been shown to be a satisfactory and preferred alternative to in-person follow-up after general surgical discharge, with a majority of patients expressing greater satisfaction with VOPCs [22].

Internet-enhanced continuity of care programs, such as those utilizing mobile medical platforms for pediatric strabismus surgery, have significantly reduced postoperative complications, improved children's quality of life and mental health, and enhanced parental satisfaction and engagement [3]. In liver transplant patients, a THMP, including electronic tablets and Bluetooth-enabled vital sign monitoring, daily questions, and video education, was associated with significantly fewer readmissions and improved quality of life [23]. Tele-education provides instruction and teaching via telecommunication, particularly valuable in resource-limited regions, saving time and costs while improving the breadth and standard of medical services [24].

Technological challenges

Challenges with technological interventions include the need for interpreter services, concerns about stable internet connections, the requirement for user-friendly platforms, and potential “digital divides” where audiovisual satisfaction is higher among higher-income patients [26]. The lack of a physical examination remains a limitation of phone calls and a barrier to broader telemedicine adoption for some follow-up scenarios [19,26]. Electronic health records (EHRs) facilitate data review in real time, helping care teams respond to alerts and manage patient issues [23]. However, the literature does not provide specific details on the use of artificial intelligence (AI) for predicting outcomes or improving the management of postoperative care beyond general mentions of AI training and similar technologies [29]. Strategies to enhance SCOC in settings with limited resources emphasize cost-effective and scalable solutions. As discussed, telemedicine is highly beneficial for reaching patients in rural areas, war zones, and correctional institutions where access to major medical facilities is limited. Its ability to reduce travel and clinic appointment needs also contributes to cost savings [24].

Future directions and recommendations

Future SCOC initiatives will likely continue to integrate advanced technology and refine care models through technological advancement. This includes the development of the following new mobile applications for patient care and education: (i) advanced visualization capabilities, such as three-dimensional telesurgical viewing and virtual interactive presence, which can enhance the operating surgeon's experience and allow teaching surgeons to digitally guide a surgery in progress [24], (ii) Bluetooth pills for medication adherence, and (iii) sweat technology for remote electrolyte monitoring, reducing the need for traditional lab draws [23]. The concept of “home hospital status” for patients with complications, with teams providing IV fluids, nasogastric tube placement, lab draws, and IV antibiotics at home, potentially keeping patients out of the hospital even with existing complications [23]. There is a critical need for more rigorous multicenter longitudinal studies to assess the long-term impact of SCOC interventions on patient outcomes. Such studies should include standardized multimodal care pathways and better characterize the impact of specific elements within these pathways. Include cost-effectiveness analyses to measure the financial benefits of interventions, which is currently a noted limitation in many studies [19,23,29]. Follow-up periods can be expanded to over a year to capture long-term effects and strengthen scientific validity [5].

Implications for practice

To implement effective SCOC, healthcare providers should consider comprehensive discharge planning and utilize programs with comprehensive checklists or designated medical professionals (e.g., nurses) to ensure all components of the treatment plan are achieved and communicated, provide patient-focused, procedure-specific education that begins early (even preoperatively) and continues post-discharge to improve patient comprehension and self-management [19,23], and implement timely follow-up mechanisms, particularly for patients predisposed to complications. While phone calls alone may not consistently reduce readmissions, specialized home visits by familiar providers have shown effectiveness. APPs can be systematically integrated into surgical teams to provide early, structured outpatient follow-up, which can lead to earlier complication identification and proactive management, preventing avoidable readmissions and improving rescue rates [29]. Telemedicine and VOPCs should be utilized for simple consultations and straightforward follow-up evaluations to reduce patient burden, free up clinic appointments for new patients, and improve patient satisfaction [22,26]. Clinical data exchange, inter-facility communication, and telemedicine platforms should be used to minimize care fragmentation, especially when patients are evaluated at different hospitals [20,21]. Standardized peer mentoring programs should be implemented to provide psychosocial support and reinforce discharge instructions, which can significantly reduce patient anxiety and encourage early communication of symptoms [25]. ERAS protocols should be adhered to, to improve postoperative recovery and shorten hospital LOS, and can reduce 30-day postoperative mortality without increasing complications or readmission rates when compliance is high [4].

Policy implications

Healthcare policy plays a crucial role in supporting better continuity of care. Policymakers need to address long-term reimbursement for telemedicine and complex issues like multi-state licensing requirements for surgeons [26]. Lack of consistent and sufficient reimbursement by payers, including Medicaid and Medicare, limits telemedicine's feasibility, particularly in underserved populations [23]. Current reliance solely on 30-day readmission rates as a quality metric may be insufficient [12]. Policies should acknowledge that some readmissions can be beneficial, enabling early identification and management of complications. Metrics should consider out-of-system readmissions (e.g., readmissions to non-Veterans Health Administration facilities), as their exclusion can distort hospital performance rankings and prevent a comprehensive view of quality. Policies should recognize that factors outside the direct control of surgical practitioners, such as social support, insurance status, or health-related post-discharge behaviors, influence readmission likelihood [28]. The tracking of a wider range of patient-centric outcomes, such as anxiety levels, should be encouraged to better capture the overall quality of care [25].

Training and education

There is a clear need for better training for healthcare professionals on the importance and practical application of SCOC principles [5]. Tele-education offers a valuable tool for educating both new surgical trainees and experienced surgeons on new techniques, especially in settings lacking abundant resources or mentors [24]. For peer mentoring programs, structured training is essential to ensure mentors understand their role in reinforcing clinical instructions and providing appropriate psychosocial support [25].

## Conclusions

Comprehensive discharge planning, individualized patient education, timely follow-up (especially with the operating surgeon or through home visits by familiar providers), and the integration of APPs can substantially reduce readmissions. While common, follow-up phone calls alone were not consistently sufficient to reduce readmissions. Care fragmentation, particularly the absence of surgeon care continuity during readmission, has been independently associated with increased 30-day mortality and a longer time to receive necessary interventions. Technological interventions like telemedicine offer promising avenues for remote monitoring, education, and consultation, leading to improved patient satisfaction, reduced costs, and lower readmission rates across various surgical specialties. Additionally, peer mentoring programs can effectively reduce patient anxiety and facilitate earlier communication of complications, even if these result in an increase in beneficial early readmissions.

ERAS protocols have also demonstrated effectiveness in shortening LOS and improving recovery. This review reinforces the critical importance of understanding and improving SCOC as a cornerstone of patient-centered care. By bridging the gap between hospital discharge and home recovery, effective SCOC interventions (e.g., comprehensive discharge planning, individualized patient education, ERAS protocol, timely follow-up, and integration of APPs) can demonstrably improve patient outcomes, reduce burdensome readmissions, enhance patient satisfaction, and elevate overall quality of life. The multifaceted nature of continuity of care, involving clinical, informational, and relational aspects, highlights the need for integrated and coordinated efforts across the healthcare continuum.
